# In vivo rate-determining steps of tau seed accumulation in Alzheimer’s disease

**DOI:** 10.1126/sciadv.abh1448

**Published:** 2021-10-29

**Authors:** Georg Meisl, Eric Hidari, Kieren Allinson, Timothy Rittman, Sarah L. DeVos, Justin S. Sanchez, Catherine K. Xu, Karen E. Duff, Keith A. Johnson, James B. Rowe, Bradley T. Hyman, Tuomas P. J. Knowles, David Klenerman

**Affiliations:** 1 Yusuf Hamied Department of Chemistry, University of Cambridge, Lensfield Road, Cambridge CB2 1EW, UK.; 2 Department of Clinical Neurosciences, University of Cambridge, Biomedical Campus, Cambridge CB2 0QQ, UK.; 3 Department of Neurology, Harvard Medical School, MassGeneral Institute for Neuro-degenerative Disease, Massachusetts General Hospital, Charlestown, MA 02114, USA.; 4 Denali Therapeutics Inc., South San Francisco, CA 94080, USA.; 5 Dementia Research Institute, University College London, London W1T 7NF, UK.; 6 Medical Research Council Cognition and Brain Sciences Unit, Cambridge CB2 7EF, UK.; 7 Cambridge University Hospitals NHS Trust, Cambridge CB2 0SZ, UK.; 8 Cavendish Laboratory, University of Cambridge, 19 JJ Thomson Avenue, Cambridge CB3 0HE, UK.; 9 UK Dementia Research Institute, University of Cambridge, Cambridge CB2 0XY, UK.

## Abstract

Both the replication of protein aggregates and their spreading throughout the brain are implicated in the progression of Alzheimer’s disease (AD). However, the rates of these processes are unknown and the identity of the rate-determining process in humans has therefore remained elusive. By bringing together chemical kinetics with measurements of tau seeds and aggregates across brain regions, we can quantify their replication rate in human brains. Notably, we obtain comparable rates in several different datasets, with five different methods of tau quantification, from postmortem seed amplification assays to tau PET studies in living individuals. Our results suggest that from Braak stage III onward, local replication, rather than spreading between brain regions, is the main process controlling the overall rate of accumulation of tau in neocortical regions. The number of seeds doubles only every ∼5 years. Thus, limiting local replication likely constitutes the most promising strategy to control tau accumulation during AD.

## Teaser

Local replication doubles the number of tau aggregate in Alzheimer’s disease only once every 5 years, limiting the overall rate.

## INTRODUCTION

Alzheimer’s disease (AD), similar to several other aggregation-associated neurodegenerative diseases (*1*, *2*), is characterized by a progressive decline in health over the course of several years, with symptoms often only becoming apparent years after the onset of pathological changes in the brain. The processes that are believed to be of critical importance in the development of AD are the aggregation both of the β-amyloid (Aβ) proteins into plaques and of the tau proteins into neurofibrillary tangles (NFTs) (*3*). While Aβ aggregation is believed to be an important event in the development of AD, clinical symptoms, atrophy, and brain damage correlate best with the appearance of tau aggregates (*4*). Tau aggregates have the ability to self-replicate, and these replication-competent aggregates are referred to as proteopathic seeds. Once an initial seed is present, it can replicate to form a large number of new seeds. Synthetic tau filaments made from recombinant protein and filamentous material extracted from tau mouse models or AD brains have been shown to act as seeds in various model systems and initiate tau pathology (*5*–*9*). Furthermore, several mouse model systems provide evidence that seeds spread from the regions in which they are initially formed to other regions of the brain and trigger aggregation there (*5*, *10*–*12*). The molecular processes that lead to tau seed replication and spreading are not known in detail but, based on animal models, are postulated to involve aggregation, transport down axons, release, uptake, and, finally, replication in the recipient neuron.

It is believed that the patterns of location and abundance of tau NFTs observed in postmortem AD brains, which form the basis for the classification of AD into Braak stages (*13*, *14*), arise from the spread of tau seeds along well-established connections through the brain. If the rate of this spread is slow enough (*8*, *12*), and assuming that aggregation begins in a single location, it has been proposed that spreading from one brain region to the next could be a key limiting factor in disease progression (*7*, *15*). Therefore, while there is consensus that both the replication and spatial spreading of seeds occur in vivo, a key unanswered question in the study of AD in particular, and aggregation-related diseases in general, is at what rate these processes occur and how important the local replication of seeds and their spread over longer length scales, between brain regions, are for determining the time scale of human disease.

In this work, we establish a general theoretical framework to determine the rates governing tau accumulation and apply it to measurements of seed and aggregate concentrations in AD brains to determine the rate-limiting process and calculate the associated reaction rates. Our model is formulated in terms of general classes of processes and is therefore able to describe the wide range of possible mechanisms of replication and spreading in vivo and determine the effective rates of these processes. This fundamental model naturally results in two limits, where either both long-range spreading and replication or local replication alone dominates the kinetics of tau accumulation. In practice, one is unlikely to reach these limits, and we instead expect either a situation where spreading and replication are equally important or one where changes in the replication rate have a much more pronounced effect on the overall rate than changes in spreading.

**Fig. 1.**
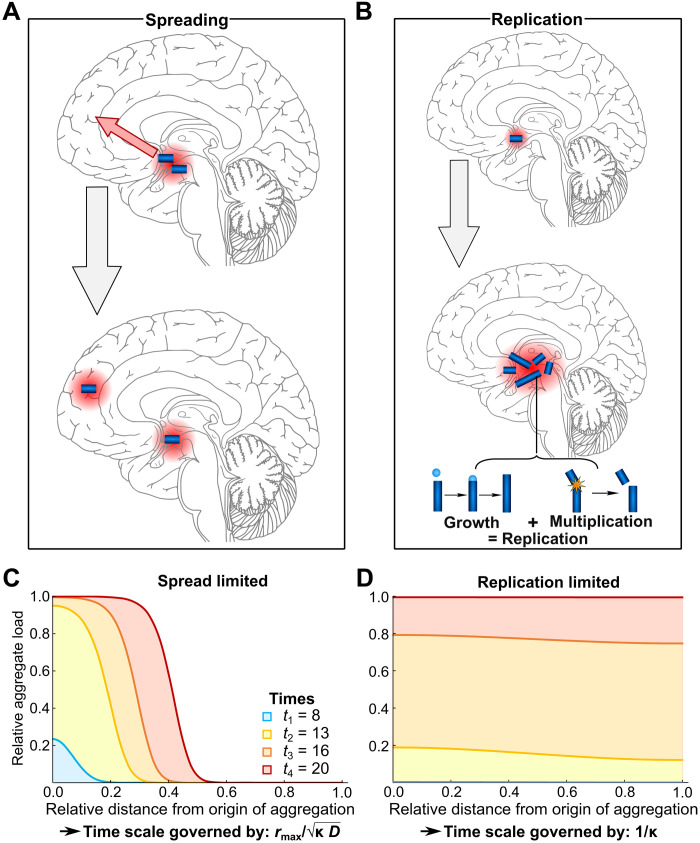
Illustration of the key processes in tau aggregate formation. (**A**) Spreading denotes spatial relocation of an existing aggregate. (**B**) Replication, the localized production of new seeds from existing ones, is composed of subprocesses that naturally fall into two categories—those that increase seed number (multiplication) and those that increase the size of a given seed (growth). (**C** and **D**) Seed load as a function of time and distance, from the solution of Eq. 1 in the two limits. In (C), the spread is slow [*D*/κ = 0.00025 (unit length)^2^] and the system is spreading-limited; in (D), the spread is fast [*D*/κ = 0.025 (unit length)^2^] and, thus, the system is replication-limited (see Materials and Methods for an approximate conversion from these reduced units).

To allow precise statements to be made on the basis of our models, we give here clear definitions of a number of terms as used throughout this work (see also Fig. 1, A and B): Replication is the process by which one seed can grow and multiply to become two (or more) seeds, which are both capable of further replication. Its subprocesses naturally fall into two categories, growth and multiplication. Growth processes increase the size of a given seed by the addition of more proteins. Multiplication processes increase the number of growth-competent seeds and encompass a wide range of processes, from simple fragmentation of seeds to indirectly induced multiplication via other biological processes. Growth and multiplication couple together, as new seeds have to mature by growth before multiplying again. Aggregate refers to all aggregated species. Seeds are aggregates that are replication competent. Spreading is any process that results in the spatial relocation of aggregates within a cell or from cell to cell, including diffusion and any active transport processes. However, the data analyzed here are only spatially resolved to the level of different brain regions, and thus, any information about spreading extracted from these data applies only to spreading over long distances between brain regions, and when spreading is mentioned in reference to those data, it refers to this long-range spreading.

## RESULTS

### Mathematical models predict two limiting behaviors

We develop a general model by considering the different fundamental classes of processes and grouping together similar phenomena into one effective term. The level of coarse graining is dictated by the detail of the experimental data available, ensuring that the simplest model consistent with the data is used.

The fundamental model that includes both spreading and replication takes the form of a spatially dependent reaction equation (*16*, *17*)

$$\frac{\partial f(r,t)}{\partial t}=D\nabla^{2}f(r,t)+\kappa f(r,t)(1-f(r,t))$$ (1)

where *f* (**r**, *t*) is the seed concentration relative to some maximal concentration, *P*_max_, at time *t* and position **r**. *D* is an effective diffusion coefficient that determines the speed of spreading, and κ is an effective replication rate. The first term on the right-hand side of Eq. 1 accounts for spreading in the form of an effective diffusion term. This common mathematical description of transport covers a wide range of active and passive processes in addition to simple diffusion (*18*). The second term accounts for replication, producing an initially exponential increase in seeds, which levels off as the maximal seed concentration is approached. The introduction of this maximal concentration is motivated by basic physical considerations and experimental observations of such a limit.

Two limiting regimes naturally emerge from the description in Eq. 1, which we refer to as the replication-limited and spreading-limited cases (see Fig. 1; for mathematical details, see section S2.1). In a replication-limited regime, the overall time scale of the reaction is determined exclusively by the replication of seeds, which is the case when non-negligible amounts of seeds are present throughout the reaction volume before the limit in seed concentration is reached anywhere (see Fig. 1D). This can be achieved in two ways, either by the fast spreading of an initially localized distribution or by the initial presence of a small concentration of seeds everywhere throughout the reaction volume. Experimental realizations of this latter scenario would be systems in which seeds are introduced globally, or systems in which the spontaneous formation of seeds, directly from monomers, happens at many locations throughout the brain. By contrast, in a spreading-limited regime, replication is so fast that each region reaches the maximal seed concentration before a significant amount of seeds can spread to the next region. At all times, most regions are either essentially free of seeds or at the limiting concentration of seeds. There is a clear propagation front moving through the reaction volume (see Fig. 1C). This distinction of regimes is important because different processes govern the overall time scale of aggregate accumulation depending on the regime the system is in: In the replication regime, the time scale is governed only by the replication rate, whereas in the spreading regime, named so for simplicity, the time scale is governed by both the replication rate and the spreading rate, specifically their geometric mean. The key point is that a decrease in the rate of replication always slows the overall rate of aggregate accumulation, whereas a decrease of the rate of spreading is only effective at slowing the overall process when the system is spreading-limited. Even in that limit, slowing replication is just as effective as slowing spreading. Thus, on the basis of these fundamental considerations alone, replication is always the preferable target for inhibition of aggregate accumulation from a mechanistic viewpoint. While this binary classification is helpful to characterize systems, in practice, a system is unlikely to fully fall into one limiting regime or another. Therefore, spreading-limited should be interpreted as “both spreading and replication contribute considerably to the overall rate,” whereas replication-limited means “replication is considerably more important in determining the overall rate than spreading.”

The general nature of this minimal model is valuable for providing a fundamental understanding of a wide range of systems and can be used to extract rates from experimental data, but one has to be careful in the interpretation of these parameters obtained from data. Replication includes any process by which existing aggregates trigger the formation of new ones, such as indirect replication by triggering inflammation, and not just those one may be familiar with from in vitro aggregation reactions, such as fragmentation of seeds. The spatial resolution of the data also needs to be taken into account when interpreting the meaning of the spreading term. In particular, in the context of the data analyzed here, which are spatially resolved only to the level of brain regions, spreading refers to the transfer of seeds between brain regions, and potential effects of local cell-to-cell transfer will be subsumed into an effective local replication term.

The rates of spreading and the rates of replication as well as the size of the system and, crucially, the initial distribution of seeds determine which limit the system is in. These limiting regimes are a general feature and emerge regardless of whether spread is assumed to proceed directly through three-dimensional space or along a specific axonal pathway (see sections S2.2 and S2.3).

### Accumulation rate of tau seeds in human AD is dominated by local replication at later stages

Using the ability of tau seeds to replicate, low concentrations of these replication-competent aggregates can be detected in brain samples using an amplification assay. In the study of DeVos *et al.* (*19*), we measured the seed activity in six different brain regions from 29 individuals with AD neuropathological changes from Braak I to Braak VI. We investigated regions that contained NFTs and areas that were presumed to contain synaptic projections of brain areas that had NFT. We found detectable seeding activity one or two synapses away from areas that had developed NFTs. Thus, in a given brain region, this assay finds replication-competent seeds in high concentrations at earlier Braak stages than more conventional measurements of NFTs would. Both types of data are analyzed and compared below. From these data, together with further measurements by the same technique from Furman *et al.* (*20*) and Kaufman *et al.* (*21*), as well as measurements by orthogonal methods using neuropathological approaches in human disease from Gómez-Isla *et al.* (*22*) and longitudinal in vivo tau positron emission tomography (PET) imaging, we here determine the rates and rate-determining steps of tau accumulation during AD.

**Fig. 2.**
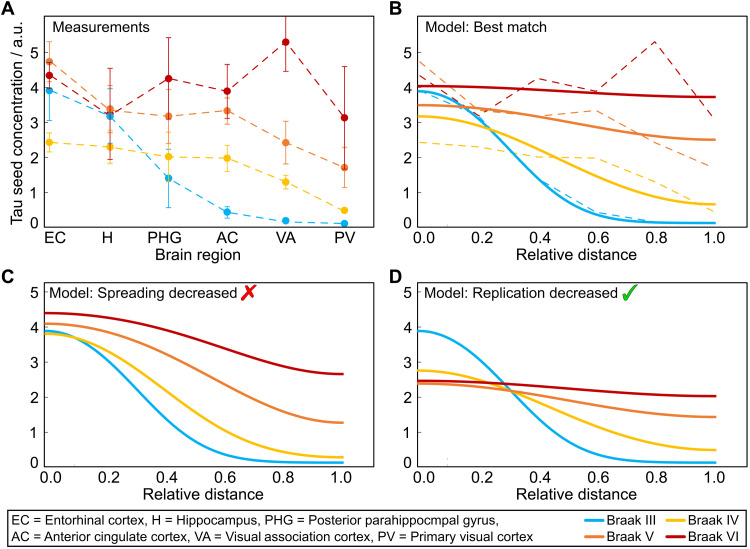
Data fits and effect of decreasing spread or replication. (**A**) Dots show the experimentally measured distribution of tau seeds, sampled in several brain regions, at different stages of the disease from DeVos *et al.* (*19*) [mean over measurements from seven (Braak III), four (Braak IV), six (Braak V), and two (Braak VI) patients; error bars are SEM]. The regions from left to right correspond to increasing distance from the EC, where aggregates first appear. a.u., arbitrary units. (**B**) Solid lines are the results of fitting Eq. 1 to the data in (A), using the data at Braak stage III as a starting point. The dashed lines represent the data, assuming that the sampled regions are equidistant along the spreading path. How the seed concentrations in the different Braak stages would change is shown for a decrease of either the spreading rate (**C**) or the replication rate (**D**) by a factor of 3. The change is much more pronounced when replication is reduced, highlighting that the system is in a replication-limited regime.

**Fig. 3.**
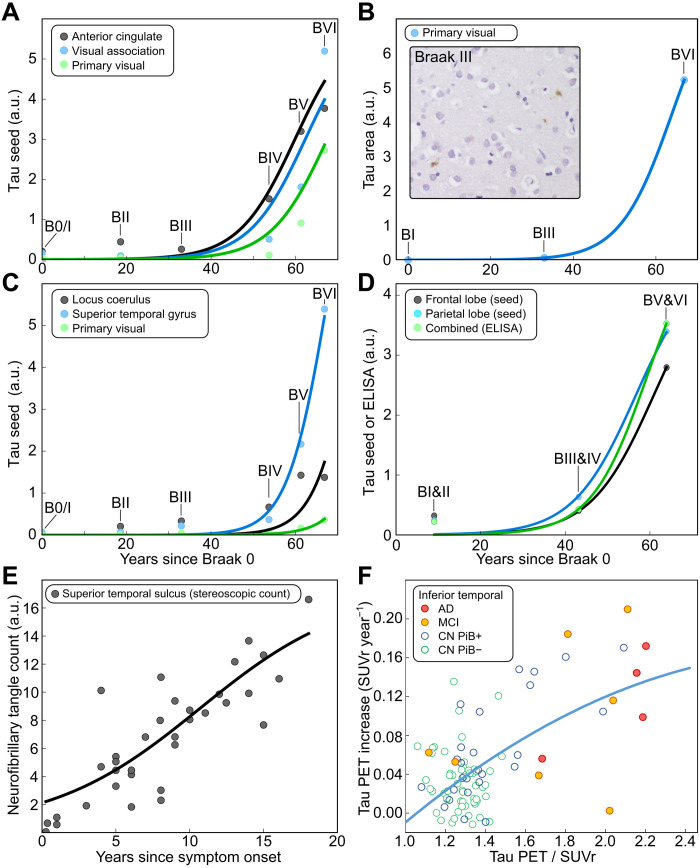
Replication rate from different datasets. To determine the replication rate κ, the solution to Eq. 1 in the replication limit was fitted to (**A**) the temporal evolution of seed concentration in the neocortical regions and (**B**) the aggregate amounts measured by AT8 staining of brain slices from the primary visual cortex (images at Braak stages 0/I, III, and VI were thresholded for AT8 response quantification, normalized by the number of cells; inset: example image at Braak stage III). (**C**) Seed measurements from Kaufman *et al.* (*21*). (**D**) Seed and ELISA measurements from Furman *et al.* (*20*). (**E**) Stereological counts of neurofibrillary tangles from Gómez-Isla *et al.* (*22*). (**F**) Longitudinal tau PET (^**18**^F-flortaucipir) measurements from Sanchez *et al.* (*24*), which determined the rate of change in tau signal over consecutive measurements approximately 2 years apart. Each data point corresponds to the rate of change plotted against the total signal in 1 of 101 individuals tested: 4 diagnosed AD, 7 with mild cognitive impairment (MCI), 27 Aβ-positive cognitively normal (CN PiB+), and 63 Aβ-negative cognitively normal (CN PiB−). These data thus measure different quantities to those shown in (A) to (E) and are hence displayed differently; however, the same model as in (A) to (E), via Eq. 5, is used to fit the data. For details of the data used, see Tables 1 and 2.

The spatial distribution of seeds at later Braak stages is shown in Fig. 2A. Three key observations guide the further analysis of these data: (i) The seed concentrations appear to increase in a concerted manner in the neocortical brain regions. (ii) Spatial inhomogeneity is most pronounced in the early Braak stages up to stage III (see fig. S1). (iii) There is a low but significant concentration of seeds even in the neocortical regions already before Braak stage III. To confirm this early presence of tau aggregates in the neocortical regions by an orthogonal method, we quantified aggregated tau in brain slices stained with the anti-tau antibody AT8 by image analysis. The data (Fig. 3B) confirm that the presence of neuritic AT8-positive pathological staining can be detected even in the primary visual cortex at Braak stage III.

**Table 1.** Summary of datasets analyzed and method used.

**Authors**	**Method**	**Data type**	**Time axis**
DeVos *et al.* (*19*)	Seed amplification	Postmortem	Time from Braak stage
This work	AT8 stain	Postmortem	Time from Braak stage
Kaufman *et al.* (*21*)	Seed amplification	Postmortem	Time from Braak stage
Furman *et al.* (*20*)	Seed amplification and ELISA	Postmortem	Time from Braak stage
Gómez-Isla *et al.* (*22*)	Neurofibrillary tangle count	Postmortem	Time since symptom onset
Sanchez *et al.* (*24*)	Longitudinal tau PET	In vivo	Real time

**Table 2.** Summary of datasets analyzed and patients measured. EC, entorhinal cortex; H, hippocampus; PHG, posterior parahippocampal gyrus; AC, anterior cingulate cortex; VA, visual association cortex; PV, primary visual cortex; LC, locus coeruleus; STG, superior temporal gyrus; FL, frontal lobe; PL, parietal lobe; STS, superior temporal sulcus; IT, inferior temporal.

**Authors**	**Disease stages**	**Brain regions**	**No. of** **individuals**
DeVos *et al.* (*19*)	Each Braak stage	EC, H, PHG, AC, VA, PV	29
This work	Braak 0, III, VI	PV	25
Kaufman *et al.* (*21*)	Each Braak stage	LC, STG, PV	247
Furman *et al.* (*20*)	Braak stages*	FL, PL	47^†^
Gómez-Isla *et al.* (*22*)	Symptomatic	STS	34
Sanchez *et al.* (*24*)	Any	IT	101

*Braak stages are grouped as I and II, III and IV, and V and VI.

†Not all individuals measured in all regions; for further details, see (*20*).

The spatial distribution of seeds shows that the later stage behavior appears qualitatively distinct from that of the very early Braak stages, during which most seeds are found in the entorhinal cortex (EC), hippocampus, and posterior parahippocampal gyrus (PHG). During these early stages, high seed concentrations appear to be confined to the EC and hippocampus, and the concentrations in all other regions remain relatively low. After Braak stage III, the seed distributions change much more quickly with time. Notably, the deposition of amyloid in the neocortex and the appearance of AD-like symptoms also tend to occur only in later Braak stages. For the model presented here, we thus focus on the faster, disease-associated phase of the disease, i.e., post-Braak stage III.

We represent this situation in our model by imposing the following initial condition in Eq. 1: At time 0, the concentration of seeds is modeled on the distribution measured in Braak stage III. To make the link to Eq. 1, the measured brain regions are represented by equally spaced locations in our model. This choice of effective distance reflects the fact that the number of synaptic connections to be crossed to go from one region to the region associated with the next higher Braak stage is similar for all regions. In reality, the graph of connections between these regions is of course considerably more complex, but this simple geometry is sufficient for illustrating the effects of spreading and replication. The effect of changes of this geometry is discussed in the Supplementary Materials. To obtain a measure for time, we convert from Braak stage to the length of time for which each stage lasts based on the extensive dataset by Braak *et al.* (*14*) (for details, see Materials and Methods) (*23*). As the switch between the relatively constant distribution of the initial stages and the global increase of the later stages happens between Braak stages III and VI, we choose the start time to lie between Braak stages III and VI. This value is optimized, but the time between consecutive Braak stages is set based on the data from Braak *et al.* The replication rate is set to κ = 0.14 years^−1^, the value obtained from combining the results of the analysis of all datasets below. The effective diffusion constant *D* and the maximal concentration in measured units, *P*_max_, are optimized to best fit the data.

The best fit with experiment, shown in Fig. 2B, is achieved when the system is mostly replication-limited. This is the case both because the initial distribution at Braak stage III is already rather spread out, with significant seed concentrations in all regions, and because the spreading rate appears relatively fast.

To further highlight the importance of replication over spreading in this system, we model how the seed distributions in Fig. 2B would change over time if the rate of either spreading (Fig. 2C) or replication (Fig. 2D) was decreased by a factor of 3. While a decrease of spreading results only in minor changes, a decrease of replication would clearly be effective at slowing the overall accumulation of tau seeds; thus, the system is replication-limited (for further details, see section S2.2). This means that the overall rate of tau accumulation is dominated by the rate of replication, post-Braak stage III, and that inhibiting replication will slow down the overall process the most. It does not mean that no spreading is taking place, or that all brain regions should experience the same aggregate load at the same time.

### Tau seeds in AD have a doubling time of approximately 5 years

Having thus established that the overall rate is dominated by local replication, we can extract quantitative information from the measurements of seeds in AD brains in the form of an effective replication rate. We fit the approximate solution of Eq. 1 in the replication-limited case (see eq. S4) to the increase in seed concentration over time, measured by a range of methods, in a number of different neocortical regions (see Fig. 3). For simplicity, Fig. 3 shows the least-squares fits to the median, but Bayesian inference on all data points was performed to determine the effective rate constant of replication as κ ≈ 0.17 ± 0.05 years^−1^ (errors are 1 SD; for details, see Materials and Methods). This corresponds to a doubling time, *t*_2_, i.e., the time taken to double the number of seeds, of approximately 4 years, where $t_{2}=\frac{\text{ln}\;(2)}{\kappa }$.

Our analysis of additional seeding data from Furman *et al.* (*20*) and Kaufman *et al.* (*21*) yields rates consistent with our data of κ ≈ 0.2 ± 0.1 years^−1^ (*t*_2_ ≈ 3.5 years) and κ ≈ 0.08 ± 0.02 years^−1^ (*t*_2_ ≈ 9 years), respectively (see Fig. 3, C and D). In addition, we also analyze data obtained by other measures of tau accumulation: (i) staining of brain slices by an anti-tau antibody, AT8 (see Fig. 3B), followed by quantification by automated image analysis; (ii) quantification of aggregated tau by enzyme-linked immunosorbent assay (ELISA), which was performed in addition to seed amplification measurements by Furman *et al.* (*20*); (iii) stereological techniques to quantify the number of NFT in the superior temporal sulcus (high order association region) for individuals who had passed away a known number of years since developing symptoms of AD [this study was performed by Gómez-Isla *et al.* (*22*) who found an apparent linear relationship between the number of accumulating tangles and the duration of disease since symptom onset]; and (iv) longitudinal tau PET in human participants with varying levels of amyloidosis and clinical impairment, which was performed by Sanchez *et al.* (*24*), evaluating longitudinal change in tau PET radiotracer-specific binding and measuring change rates in individuals over the course of approximately 2 years.

While these measurements may quantify different forms of tau than the seeding assay, the same equations can be applied, and we find replication rates of κ ≈ 0.17 years^−1^ (ELISA, *t*_2_ ≈ 4 years), κ ≈ 0.20 years^−1^ (AT8, *t*_2_ ≈ 3.5 years), κ ≈ 0.22 years^−1^ (stereological counting, *t*_2_ ≈ 3 years), and κ ≈ 0.19 years^−1^ (PET, *t*_2_ ≈ 3.5 years). Crucially, this close agreement highlights that our findings are consistent across a variety of assays used to quantify aggregated tau. Combining the data (see Materials and Methods) yields an average rate of κ ≈ 0.14 years^−1^ corresponding to a doubling time of ∼5 years. Moreover, while the time axis in the seed replication and ELISA data was obtained from the average time between Braak stages, the time in the stereological neuropathological counting data and the PET data corresponds to real time elapsed since the first occurrence of symptoms or time between PET measurements, respectively. This combination of a variety of methods to determine both the seed or aggregate concentration and the time confirms the robustness of our conclusions about the doubling time. Moreover, the fact that the rates obtained from data that quantify replication-competent seeds and those that quantify mature aggregates (such as AT8 staining) are comparable implies that the appearance of these types of species is likely to be governed by similar processes. Notably, all methods yield a markedly low rate of replication, with a doubling time of several years, which implies that a large number of seeds and aggregates have to form initially to achieve the high final concentrations. Given the above rates, an increase by approximately 100- to 1000-fold is expected in the few decades of the disease. By contrast, even the number of large aggregates in the final stages of the disease is only a few orders of magnitude less than the number of neurons in the brain [i.e., at least tens of millions of aggregates, corresponding to one aggregate per every hundred neurons (*22*)]. If the disease were to be initiated by few aggregates in a specific location, reaching the observed late-stage disease state would require an increase many orders of magnitude faster than that predicted with a doubling time of ∼5 years. This implies that at Braak stage III, there are already many aggregates widely distributed across the brain.

**Fig. 4.**
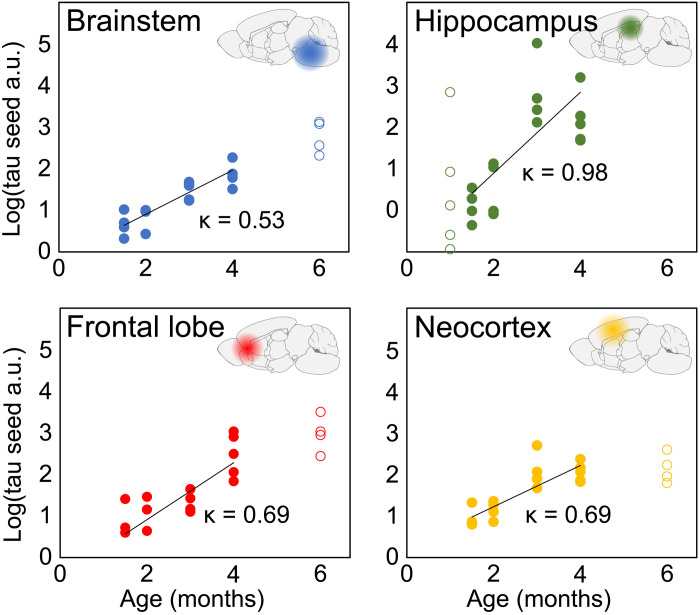
P301S mouse model is also replication-limited. The logarithm of tau seed concentration in P301S transgenic mice, measured by fluorescence resonance energy transfer (FRET) flow cytometry, at several time points and locations throughout the brain. The data (filled and open circles) were obtained from Holmes *et al.* (*25*). The solid line is a straight line fit in logarithmic space. For the fitting, only times were taken into account for which data exist in all brain regions (filled circles) to avoid biases.

To further compare these results with a common model system of tau pathology, we analyzed data from P301S mice, measured by Holmes *et al.* (*25*) with the same seed amplification assay. Mouse models allow the minimization of variation between different animals, in particular concerning the time of onset of disease, and thus represent a well-controlled model system. Two features of these data are noteworthy: First, the increase in seeding activity is exponential during early disease, a hallmark of the autocatalytic feedback loop associated with seed replication (*26*, *27*). Analyzing the data up to 4 months, we determine the rate of replication, κ = 0.6 months^−1^, which corresponds to a doubling time of approximately 2 weeks (in the brainstem, neocortex, and frontal lobe). Second, there is no evidence of a delayed initiation of the aggregation reaction in any brain regions (Fig. 4), suggesting that there are few spatial inhomogeneities and that the system is again replication-limited. Therefore, aggregation either begins at many locations throughout the brain, as might be expected in a transgenic overexpressing animal, or begins at a small number of locations but spreads much faster than the time scale of the disease. In either scenario, local replication dominates the kinetics of tau seed accumulation and proceeds at a rate orders of magnitude faster than in humans.

## DISCUSSION

### Replication in AD is orders of magnitude slower than in mouse models or in vitro

**Fig. 5.**
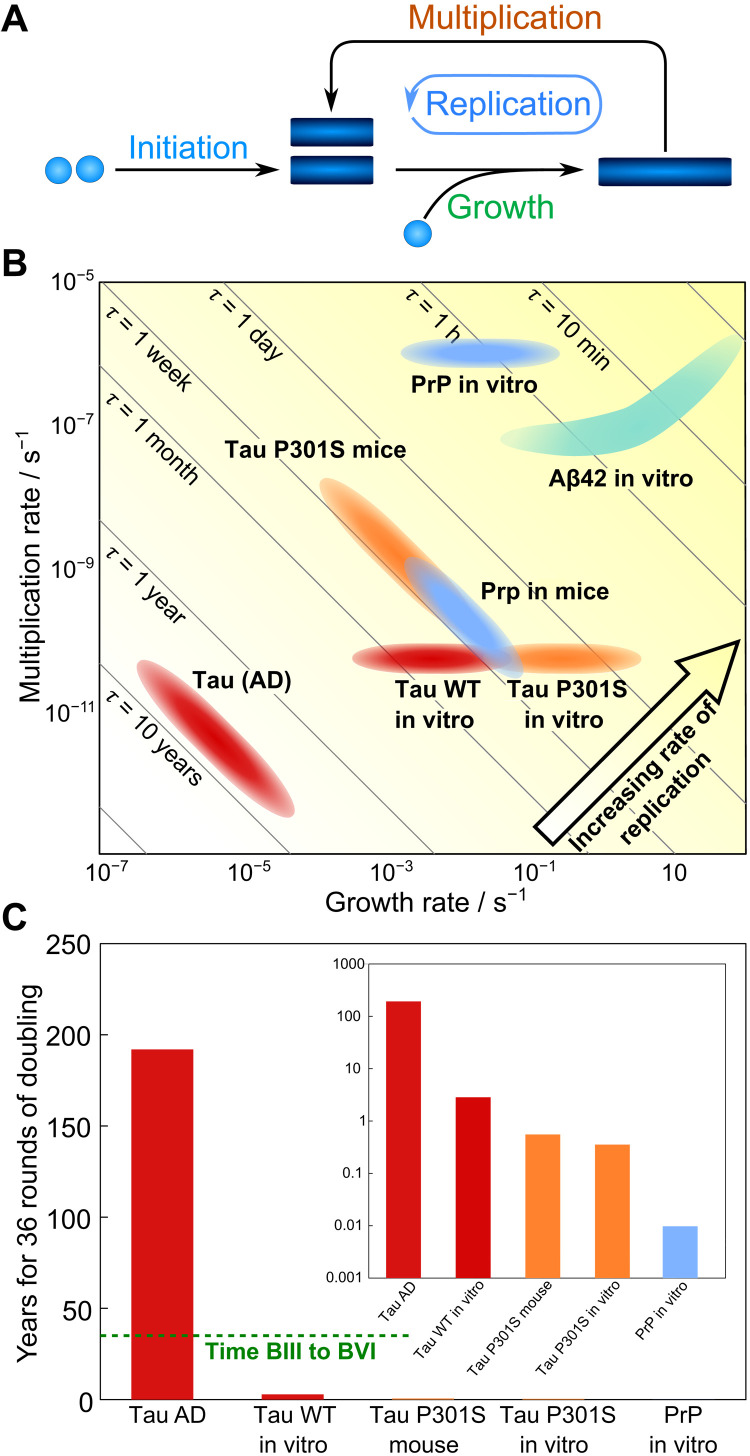
Rate comparison with other systems. (**A**) Key steps of aggregation reactions illustrating how growth and multiplication combine to lead to seed replication. (**B**) Comparison of the rates of tau seed growth and multiplication determined in AD brains here, with the rates predicted at concentrations between 100 nM and 10 μM from in vitro experiments (*28*), as well as the rates for both PrP (*39*) and Aβ42 (*40*) replication in vitro at the same concentrations (for details, see Materials and Methods). Diagonal lines show the order of magnitude of the doubling time, i.e., points along the diagonal lines have the same doubling time and replication rate. (**C**) Time required to produce 70 billion seeds from one seed (i.e., time for 36 rounds of doubling) for comparison with disease time scales; the dashed green line shows the average time that passes between Braak stages III and VI. Only the two slowest systems, wild-type (WT) tau, are visible on a linear scale; inset shows time on a logarithmic axis.

Having extracted the rates of replication tau seeds in AD, they can now be compared to the rates determined in mouse models or in the in vitro aggregation of tau, the prion protein (PrP), and Aβ42 (Fig. 5B). To compute the relevant range of rates from the in vitro measurements of tau aggregation, we assumed that the monomer concentration was between 100 nM (estimate for monomeric tau in solution) and 10 μM (estimate for total monomeric tau) (*28*, *29*). The differences between the individual systems are so large that they remain significant even given this uncertainty in the monomer concentration. The in vitro measurements of tau aggregation used here were performed with recombinant tau, which is believed to be considerably less aggregation prone than the phosphorylated forms encountered in vivo, so the decrease in rate going from in vitro to in vivo may be even larger than shown here. We illustrate the biological meaning of the replication rates in the different systems by showing how long 36 rounds of doubling (producing ∼70 billion seeds from one) would take (Fig. 5C). Measurements of the average size of tau seeds allow us to dissect the replication rate in vivo derived above into the contributions from growth and from multiplication (for details, see the Supplementary Materials) by using the fact that the replication rate κ is determined by the product of growth and multiplication, whereas the average size μ is determined by their ratio

$$\begin{array}{c} \kappa =\sqrt{k_{\text{growth}}k_{\text{mult}}},\\ \mu =\sqrt{\frac{k_{\text{growth}}}{k_{\text{mult}}}} \end{array}$$ (2)

**Fig. 6.**
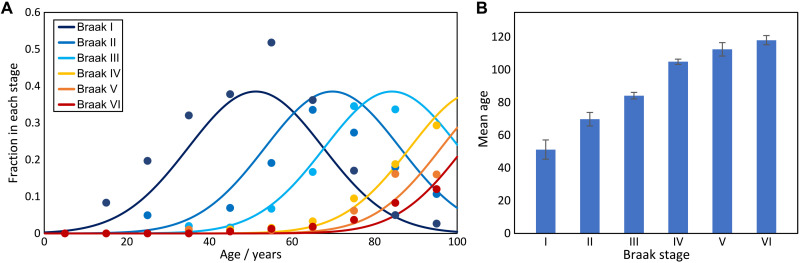
Converting Braak stage to time. (**A**) Fractions of individuals in a particular Braak stage for a given age. Data from Braak *et al.* (*14*). All individuals below Braak stage I have been grouped together into one group (not shown), whereas the remainder are as classified by Braak *et al.* The distributions of Braak stage I and above are fit to Gaussians, where the magnitude and the SD of the Gaussians are global parameters determined by the distributions of stages I to III, and only the midpoint is a free parameter for all stages. (**B**) Results of the fits; error bars are 95% confidence intervals on the mean age for each stage.

The rates of both growth and multiplication are shown for the systems analyzed here and compared to the in vitro aggregation of tau, the PrP, and Aβ42 (Fig. 5B). As can be seen in Fig. 5B, the replication rates of both PrP and Aβ42 are over an order of magnitude faster than those of tau in vitro or in mice, and tau replication in AD is even slower by a further two orders of magnitude than in vitro.

Notably, while wild-type tau replication in AD proceeds about two orders of magnitude slower than in vitro, the aggregation rate of P301S tau in vitro is comparable to replication rate of the same mutant in P301S mice. In other words, the relative decrease of the rate measured in vivo is much less pronounced for P301S mice than for human AD when compared to the rate measured for the same protein mutant in vitro. This observation may indicate that the mouse model lacks some of the mechanisms that inhibit tau aggregation in humans, especially in the setting of many-fold overexpression of a protein that has high aggregation potential. Moreover, neurons may be more effective at preventing replication when it occurs more slowly so that protective mechanisms are less easily overwhelmed. By contrast, we recently determined the replication rate of prions in mice (*27*) and found that it proceeds much faster in vitro than in vivo, indicating that in other mouse models, the effect of processes to inhibit aggregation in vivo can be much more pronounced than what we find in the mouse model analyzed here. Notably, the replication rate of prions in mice determined by Meisl *et al.* (*27*) is very similar to that of tau in P301S mice determined here. This similarity may be due to the fact that the choice of the mutant and the level of protein expression is optimized for a life span of several months in the mouse models. These observations highlight the importance of a quantitative approach to enable comparison at all levels of a system, from in vitro aggregation over in vivo model systems to human disease.

In humans, the significantly lowered rates of both growth and multiplication highlight the importance of the mechanisms that have evolved to limit the rate of replication in living systems. The replication rate we determine here from human samples contains all these effects, from the clearance of seeds and aggregates by a variety of processes to the effects of chaperones and other molecules that reduce the rates of aggregation and prevent self-replication of existing seeds. The relative slowing of growth and multiplication may serve as a guide to determine which mechanisms of inhibition are most prominent in vivo.

### Limitations and interpretation in the context of other work

A key consideration in the interpretation of the results is the level of coarse graining imposed by the experimental data, which affects the microscopic interpretation of the effective replication and spreading rates obtained from their analysis. The spatial resolution of the data is at the level of a single brain region, so while spreading between brain regions can be recovered from these data, the effects of local inhomogeneities are subsumed into an effective replication term here. In other words, while it is clear that spreading between brain regions is not rate-limiting in the overall accumulation of tau seeds after Braak stage III, effects on length scales smaller than those resolved in the data, such as transfer between neighboring cells within one region, may be of significance. Considerations of more spatially resolved data from model systems may serve as a guide as to which local transport processes, if any, are most likely to significantly contribute to the effective rate of replication that we determine from the coarse-grained data. In model systems that contain overexpressed tau, uptake and aggregation in the recipient cell can occur very quickly (within hours to days) (*12*, *25*). Yet, injecting seed-competent tau into mice that do not overexpress tau leads to production of AT8-positive phosphotau neuronal inclusions over many months (*7*). Furthermore, Evans *et al.* (*30*) detect the efficient uptake of aggregated tau (at nanomolar concentrations) into human cortical neurons in cell culture over the time scale of hours, and McEwan *et al.* (*31*) find that human embryonic kidney (HEK) 293 cells expressing aggregation-prone tau not only take up seeds that trigger intracellular aggregation within hours but also show that there are effective mechanisms to abolish the seeding effect once an aggregate has entered the cell. Together, these results are evidence that seed-competent aggregates can easily enter cells, but that there may be mechanisms that slow their replication, in agreement with our findings here.

The finding that replication is rate-limiting from Braak stage III onward and that it proceeds quite slowly raises the question of how spatial inhomogeneities arise in the first place and how the high concentrations of seeds are created before Braak stage III. Regarding the distributions, it has been demonstrated in numerous works that functional connectivity of the brain plays a key role in determining the observed patterns of NFTs and tau PET signals (*32*, *33*), and is thus likely to also be important for the distribution of replication-competent seeds that we here use to model Braak stage III. No definite conclusions can be drawn about why the replication or de novo formation appears to be governed by different rates before Braak stage III. However, through the incorporation of data at early disease stages, of a higher spatial resolution, our models could be adapted to further explore the initial sources of aggregates and determine the importance of high de novo formation rates and potential mechanisms triggering the switch at Braak stage III. One factor that could be responsible for the observed change in behavior is the formation of amyloid, which tends to only be present after Braak stage III, and may, either by direct interaction or through indirect means such as stressing of the protein control mechanisms, alter the aggregation behavior of tau.

### Broader implications of findings

By applying chemical kinetics to in vivo data, we were able to describe the spreading and replication of seed aggregates in the brain. We define two qualitatively distinct regimes, in which different processes limit the speed of overall tau seed accumulation. In particular, we find that a decreased rate of replication of seeds always slows the overall progression, whereas a decreased rate of spreading only does so under certain circumstances. Using data from human AD brains, we find that the process of tau seed accumulation is dominated by the local replication of seeds and that spreading between brain regions appears not to be a rate-limiting step after Braak stage III in the neocortical regions. The exponential increase in seed number observed in these regions is strong evidence that tau aggregates replicate autocatalytically. From these data, we are able to extract the rate of tau seed replication in human AD and find that this rate is orders of magnitude slower than that measured for purified tau in vitro, quantifying the effectiveness of innate cellular mechanisms that curtail tau seed replication. Notably, we also find that the replication rate is so slow that the high numbers of seeds present in late disease require either that many seeds are formed de novo, rather than from existing seeds, or that seed replication proceeds much faster before Braak stage III. The cause of this switch in behavior at Braak stage III is not yet established, but the change generally correlates with the appearance of amyloid deposits. The conclusions from our model, built from biosensor data and confirmed by both retrospective neuropathological analyses and prospective PET analyses, show that tau replication, rather than long-range spreading between brain regions, is likely to be the rate-limiting step during the mid and later stages of AD, which has important implications for current therapeutic strategies. We envisage that the models developed here will form the basis for determining the rate-limiting processes and quantifying their rates for a wide range of other tauopathies and aggregation-related neurodegenerative diseases in general.

## MATERIALS AND METHODS

### Immunohistochemistry

For the quantification of aggregated tau in brain slices (Fig. 3B), human brain tissue from 25 brain donors was obtained from the Cambridge Brain Bank (NRES 10/H0308/56). The donated brains had been pathologically assessed by a neuropathologist following the Consortium to Establish a Registry of Alzheimer’s Disease (CERAD) and Braak staging protocols. Cases were selected to include a variety of Braak stages. This included 7 Braak stage 0 (mean age, 64 years; range, 35 to 83), 8 Braak stage III (mean age, 84 years; range, 72 to 95), and 10 Braak stage VI (mean age, 74 years; range, 60 to 89). Deparaffinized 10-μm sections of the primary visual cortex were obtained. These were subjected to antigen retrieval in 98% formic acid for 5 min followed by 4% aqueous hydrogen peroxide to block endogenous peroxidases. Sections were then rinsed with tap water and phosphate-buffered saline (PBS) before being blocked with normal rabbit serum (Dako) in PBS. Sections were then incubated with antibody to phosphorylated tau protein (AT8, 1:500; Thermo Fisher Scientific) for 1 hour. After rinsing for 5 min in PBS, they were incubated with secondary antibody (rabbit anti-mouse, 1:200; Dako) for 30 min. After rinsing for 5 min in PBS, they were incubated in avidin-biotin complex (Vector) for 30 min before being developed with diaminobenzidine (Vector). Slides were then lightly counterstained with hematoxylin. Digital images were obtained using a camera (Infinity 2, Lumenera) attached to a microscope (Olympus BX53). Images of the primary visual cortex were obtained at a magnification of ×200 to create images measuring 5.892 mm^2^. The ethics concerning the use of samples from the Cambridge Brain Bank is covered by the Neuropathology Research in Dementia (NeRD) protocol (REC: 16 WA 0240).

Images were analyzed to determine the fraction of AT8-positive pixels and the number of cells. First, the number of cells was quantified: The 8-bit RGB (red green blue) images were thresholded with an HSB (hue-saturation-brightness) filter, passing hue value between 108 and 200, saturation value between 25 and 141, and brightness value between 145 and 230. The images were then turned into a binary image and run the following ImageJ plugins in sequence: “Dilate” (to make the boundary smoother), “Fill Holes” (to remove the artefacts), “Watershed” (to divide cells that are fused to each other), and “Analyze Particles” (only cells larger than 200 square pixels and with a circularity above 0.5 were counted). Next, the tau plague area was quantified: The 8-bit RGB images were thresholded with an HSB filter, passing hue value below 44 and above 222, saturation value above 42, and brightness value below 206. The images were then turned into a binary image, and the number of pixels passing the threshold was counted as tau plague area. The tau plague area was normalized to the cell number of the image.

### Linking Braak stage to time since disease onset

The time since disease onset is required for an accurate determination of the replication rates, but in human postmortem data, the determination of such a time since onset is difficult. The age of onset varies over decades, and symptoms only appear in the later stages, meaning that samples of early disease stages are from individuals that often had not been diagnosed before their death. The disease stage is determined by inspection of postmortem brains, which allows classification into different Braak stages. Here, we attempt to link Braak stage to time since disease onset to then be able to put the measurements of seeding activity on a common time axis. We use data on the age and Braak stage of 2332 individuals, published by Braak *et al.* (*14*) (Fig. 6).

The underlying assumption required to proceed is that once the disease has begun, it progresses in much the same manner in different individuals, i.e., the differences between individuals originate mainly from the different times of disease onset, and the disease progression itself is less variable. If this was the case, one would expect the age distribution for each disease stage to have approximately the same shape but a different average age. Moreover, the mean (or in this case equivalently the median) age at each stage should be a good guide to determine the average time spent in each stage. In practice, there is evidence that the rate of progression is to some extent correlated with the age of onset (*34*, *35*); however, for the purposes of obtaining an approximate conversion of Braak stage to time, this model is sufficient, as confirmed by the analysis of two datasets that use a time axis obtained directly [Fig. 3, E and F; those from Gómez-Isla *et al.* (*22*) and Sanchez *et al.* (*24*)].

The data by Braak *et al.* (*14*) have 10 age categories, each spanning a decade between 1 and 100 years and 12 Braak substages from stage 0 to stage VI. We initially normalize the data for each age group, resulting in the probability of being in a certain Braak stage, given the age.

During early Braak stages (I to III), the distributions look Gaussian and can easily be fitted with a global SD and individual means, confirming the above assumption. From stage III onward, the fact that there are no data available for people above 100 years of age means that the distributions are cut off. To deal with this problem, we fix the magnitude and SDs of the Gaussians based on the early stages and only fit the mean for Braak stages IV to VI. The means, i.e., predicted average ages for each stage, are shown in Table 3.

**Table 3.** Predicted mean age for each Braak stage, with 95% confidence intervals and difference between consecutive stages.

**Stage**	**Mean age**	**95% CI** **(lower)**	**95% CI** **(upper)**	**Time to** **next stage**
I	51	45.3	57.1	19
II	70	65.7	73.9	14
III	84	82.2	86.2	21
IV	10 5	103.3	106.5	8
V	113	108.4	116.6	5
VI	118	115.3	120.9	–

An alternative measure for the time scale of disease is provided by Whittington *et al.* (*36*), who obtained a measure of amyloid load (rather than tau) as a function of time during AD using PET imaging. Their results also show a progressive increase of amyloid load over approximately 30 years, consistent with the time scales we here obtain for transitioning from Braak stage III to VI.

### Previously published data

We analyzed several different datasets from previously published works. Here, we provide details of which datasets were used and how they were processed. For further details on the experimental conditions, please refer to the original publications. The data from DeVos *et al.* (*19*) are those shown in their work in Fig. 3C. The data from Kaufman *et al.* (*21*) are those shown in their work in Fig. 2 (B to D) for AD. The data from Furman *et al.* (*20*) are those shown in their work in Fig. 1 (B and C) (frontal lobe and parietal lobe seed) and Fig. 4C (ELISA). The data from Furman *et al.* (*20*) are those shown in their work in Fig. 1 (B and C) (frontal lobe and parietal lobe seed) and Fig. 4C (ELISA). For the datasets from these three works, DeVos *et al.* (*19*), Kaufman *et al.* (*21*), and Furman *et al.* (*20*), our plots show log-space averages for clarity, but the fits are performed on all data points as shown in the original works. The data from Gómez-Isla *et al.* (*22*) are those shown in their work in Fig. 5A. The details of the fitting of these data are discussed in the following. The PET data from Sanchez *et al.* (*24*) are discussed separately below.

### Fitting to obtain replication rates

To obtain the effective replication rate, we use a rescaled version of eq. S4, the solution for *f*(**r**, *t*) in the replication-limited regime, to describe the measured values, *S*(*t*)

$$S(t)=\alpha f(r,t)=\alpha \frac{f_{0}e^{\kappa t}}{1-f_{0}+f_{0}e^{\kappa t}}$$ (3)

where α is the proportionality constant that converts the fraction of seeds, *f*(**r**, *t*), to the measured quantity (e.g., intensity of AT8 response and ELISA signal). The dependence on position **r** has disappeared because we are in the replication-limited regime. This is the solution to the logistic differential equation, whose links to the description of aggregation kinetics we discuss in detail in the study of Meisl *et al.* (*37*). For clarity, we show least-squares fits to the median of the data points (Fig. 3), but to obtain the more accurate values and error bars quoted in the text, we use Bayesian inference on the individual measurements, assuming normally distributed noise, with the SD fixed from the SD of repeat measurements. We assumed a flat prior for α and a 1/*x* prior for κ and *f*_0_. The bounds for the initial fraction of seeds, *f*_0_, were chosen to be between 10^−10^ and 0.01, corresponding to there being on the order of one seed per brain and to there being 1% of the final seed concentration at the beginning of the disease, respectively. We believe that these are very generous bounds, and any values outside them are very unlikely. Where available, the measurements for each brain region were analyzed separately, and the resulting posterior was marginalized over α and *f*_0_, yielding a posterior distribution for κ. For datasets with more than one brain region, these marginalized posteriors were then combined to give an overall posterior for κ, for the entire dataset. We furthermore combined the posteriors of all experiments (different datasets of seed measurements, AT8 quantification, ELISA, and stereoscopic counting) to yield an overall value for κ, which we use to represent tau in AD in Fig. 5. This value can be interpreted as the value of κ most consistent with all data recorded by all different methods.

### PET data and analysis

Unlike the other datasets analyzed, which had only a measure of the time since disease onset, the PET data from Sanchez *et al.* (*24*) instead provide a measure of tau amounts at two time points in the same patient, separated by approximately 2 years, giving truly longitudinal data. We used the TAU PET ^18^F-flortaucipir (FTP) signal, given as standardized uptake value ratio (SUVr) and normalized to cerebral white matter, from 80 to 100 min (*38*). The data used are partial volume–corrected SUVr values of 101 individuals [4 diagnosed AD, 7 with mild cognitive impairment, 27 Aβ-positive cognitively normal, and 63 Aβ-negative cognitively normal; for definitions of these classes, see Sanchez *et al.* (*24*)]. Further experimental details and information on how the region-specific SUVr values were determined can be found in the study of Sanchez *et al.* (*24*).

The analysis of the PET data was performed by assuming that the tau PET signal measures the total amount of tau, which follows Eq. 3 and includes an additive baseline signal. The resulting equation is

$$S(t)=\alpha \frac{f_{0}e^{\kappa t}}{1-f_{0}+f_{0}e^{\kappa t}}+b$$ (4)

where the parameters are as defined in Eq. 3; *S*(*t*) denotes the measured SUVr value, and the additional parameter *b* accounts for the nonzero baseline in signal *S*(*t*). Therefore, this equation describes a sigmoidal between *b* and α + *b*. The availability of two consecutive measurements allows the determination of an annual rate of increase *r*, which we use here as an estimate for the time derivative, r≈dS(t)/dt. Differentiating Eq. 4 yields an expression for *r*

$$\text{d}S(t)/\text{dt}=r=\frac{\kappa }{\alpha }(S-b)(\alpha -(S-b))$$ (5)

which, as expected, resembles the original logistic differential equations for a shifted and rescaled function *S*. In practice, we approximate *S* by the PET signal averaged over the two consecutive measurements. Figure 3 shows a plot of *r* against *S*, with a fit of Eq. 5. For the fits, we enforce an upper bound of α < 4, i.e., the hypothetical maximum SUVr value at the plateau of tau aggregate concentration is less than 4 units above the baseline, which is well beyond the highest measured signal and therefore this value constitutes a conservative upper bound. The other two fitting parameters κ and *b* are required to be positive but otherwise allowed to vary freely. We obtain κ = 0.17 years^−1^ and thus a doubling time of approximately 4 years, with *b* = 1.06 and α = 4. A relaxation of the upper bound on α leads only to minor improvements in the fits and a slight decrease in κ.

### Comparison of in vivo and in vitro rates

In vitro, we have control over the concentrations of the reaction species and are therefore usually able to determine rate constants, rather than just rates, which, in turn, allows us to extrapolate to predict the rates at different concentrations. Given the rate constants from previously published work, we here evaluate the rates at a range of monomer concentrations to obtain a range of relevant values for comparison with the rates measured in vivo. We estimate the relevant range of tau concentrations in AD to be 100 nM to 10 μM, and thus, we evaluate the rates based on in vitro measurements in this range of concentrations. We also evaluate the rates of PrP and Aβ aggregation in this range to provide a reference on relative speed compared to other proteins. The in vitro experiments, in which the rate constants used here were determined (*28*, *39*, *40*), were performed at micromolar concentrations and are thus in the relevant range, making errors from extrapolation small. The region predicted for Aβ42 in Fig. 5 is curved because its multiplication rate depends on concentration. While these in vitro rates are determined for the aggregation of the purified protein, in vivo aggregation is influenced not only by the presence of various other compounds, such as chaperones, that affect the aggregation but also by modifications to the proteins, such as phosphorylation. The effective rates we determine here therefore include all of these factors, quantifying their combined effect.

### Dissecting multiplication and growth

It can be shown that very generally (*37*), for a growth multiplication–type mechanism, the overall rate of increase in aggregates is given by

$$\kappa =\sqrt{k_{\text{growth}}k_{\text{mult}}}$$ (6)

where *k*_growth_ and *k*_mult_ are the rates of growth and multiplication, respectively. Moreover, the average size of the aggregates, μ, in number of monomeric units, is given by

$$\mu =\sqrt{\frac{k_{\text{growth}}}{k_{\text{mult}}}}$$ (7)

Therefore, given the overall rate, κ, and the average size, μ, one can determine the rates of growth and multiplication by

$$\begin{array}{c} k_{\text{growth}}=\mu \kappa \\ k_{\text{mult}}=\frac{\kappa }{\mu } \end{array}$$ (8)

Jackson *et al.* (*41*) measured an average length of tau fibrils of 176 nm in P301S mice. Given a β-sheet separation of 0.47 nm and assuming that there are two tau monomers per layer of the structure (*42*), 176 nm corresponds to 750 monomers. To account for the fact that Jackson *et al.* (*41*) measured these sizes in P301S mice, while Fitzpatrick *et al.* (*42*) analyzed fibrils from AD, we give a conservative range of likely μ to be between 100 and 10000 tau monomers, with the likely size on the order of 1000 monomers. We use the same range for both P301S mice and AD.

### Estimates of effective diffusion constants

In section S2.1, we show that for systems with a compact initial distribution, the switch between the replication-limited and spreading-limited regimes occurs approximately when $D/\kappa =0.0025\;r_{\text{max}}^{2}$. To obtain an approximate value for the critical diffusion constant at which the switch from one regime to another occurs in the context of tau seeds in AD, we set *r*_max_ = 10 cm and κ = 0.14 years^−1^, which yields ≈10^−13^ m^2^s^−1^. However, this value assumes compact initial distributions; the more spread out the initial distribution is, the less important the contribution from spreading becomes. In the extreme case of a uniform initial distribution, no spreading whatsoever would be necessary. Diffusion coefficients for proteins are generally on the order of 10^−11^ m^2^s^−1^. Therefore, considering the fact that the effective diffusion constant we calculate here is for seeds, which are likely much larger than individual proteins and that it may include both active transport processes and membrane crossing, values either side of this critical value of *D* ≈ 10^−13^ m^2^*s*^−1^ are entirely reasonable.

The best match to the data from AD brains (Fig. 2) is achieved for $D/\kappa =0.1\;r_{\text{max}}^{2}$. For *r*_max_ = 10 cm, this would correspond to 4 × 10^−12^ m^2^ s^−1^, but this value should be considered only a rough order of magnitude sanity check rather than a quantitative result. It depends both on the choice of *r*_max_ and on the way in which we have arranged Braak stage brain regions along a linear path, evenly spaced. Alternatively, to avoid the assumption of a specific length scale, we can express the length in terms of number of crossings between brain regions, assuming that there are six regions corresponding to each Braak stage. In that case, we obtain ≈0.5 (regions)^2^ year^−1^. Thus, while we can obtain accurate results for the replication rate κ because it is dominated by local effects rather than the spreading rates and connectivity, a more realistic representation of brain geometry would be required for an accurate estimate of *D*.

## Supplementary Materials

### This PDF file includes:

Sections S1 to S3 Figs. S1 to S7

## Notes

View/request a protocol for this paper from *Bio-protocol*.
