# Supplementary material for: In vivo rate-determining steps of tau seed accumulation in Alzheimer’s disease
Source: Sci Adv. 2021 Oct 29;7(44):eabh1448. doi: 10.1126/sciadv.abh1448 (PMC8555892; doi:10.1126/sciadv.abh1448)
Supplement: Supplementary file 1 — Sections S1 to S3 Figs. S1 to S7 [file sciadv.abh1448_sm.pdf]

Science Advances
AAAS

Supplementary Materials for

In vivo rate-determining steps of tau seed accumulation in Alzheimer's disease

Georg Meisl, Eric Hidari, Kieren Allinson, Timothy Rittman, Sarah L. DeVos, Justin S. Sanchez, Catherine K. Xu, Karen E. Duff, Keith A. Johnson, James B. Rowe, Bradley T. Hyman*, Tuomas P. J. Knowles*, David Klenerman*

*Corresponding author. Email: bhyman@mgh.harvard.edu (B.T.H.); tpjk2@cam.ac.uk (T.P.J.K.); dk10012@cam.ac.uk (D.K.)

Published 29 October 2021, *Sci. Adv.* **7**, eabh1448 (2021)
DOI: 10.1126/sciadv.abh1448

This PDF file includes:

Sections S1 to S3
Figs. S1 to S7

1 Additional data

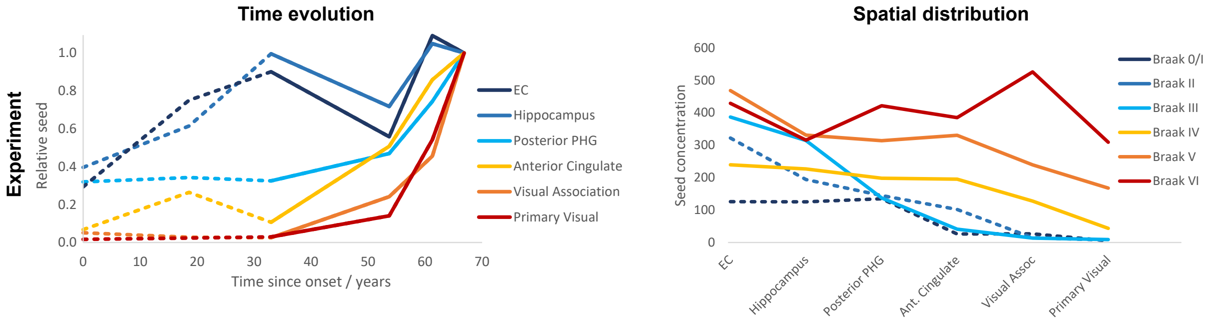

Figure S1: Full data from DeVos et al. [19], the main text shows only Braak stages 3 onwards (solid lines).

Sensitive tau seed measurements in mouse models. Utilising the ability of tau seeds to replicate, Holmes et al. [25] developed a sensitive assay for the determination of tau seeds by FRET Flow Cytometry and measured the seeding activity of tau aggregates in several brain regions of P301S transgenic mice, at several times up to one year of age. The data were extracted from Holmes et al. [25], Figures 1c and 5 in their work. The data in the former was used to obtain a calibration curve, which was then used to convert the data from the latter from FRET intensity to seed concentration. The data and fitted calibration curve are shown in Fig. S2. Several of the data points in Holmes et al. figure 5 lie outside this calibration range, and were thus excluded from Fig. 4. The data are fitted only up to 4 months as the hippocampus and frontal lobe data at later times are outside the calibration region. The inclusion of all data up to 12 months, possible only for the brainstem and neocortex, results in a lower average rate. Thus fitting all data available for each region would introduce a bias for the brainstem and neocortex. Our

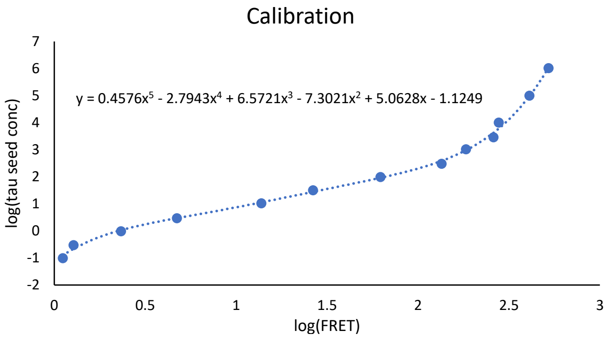

Figure S2: Data from Figure 1c in Holmes et al. [25] (dots), used to obtain a calibration curve (dashed line) that allows conversion of FRET intensity to tau seed concentration. The fifth order polynomial used is given in the figure.

fits of the data up to 4 months yield comparable replication rates for the brainstem, neocortex and frontal lobe, and a replication rate which is almost a factor of 2 higher for the hippocampus. However, given the noise of the data in the hippocampus, this deviation is not significant.

2 Theoretical Models

2.1 Reaction diffusion equation and the spreading-limited and replication-limited regimes.

The equation describing spatially-dependent aggregation is:

$$\frac{\partial P(\mathbf{r},t)}{\partial t} = D\nabla^2 P(\mathbf{r},t) + kP(\mathbf{r},t)(P_{\max} - P(\mathbf{r},t)), \tag{S1}$$

where $P(\mathbf{r},t)$ is the aggregate concentration at time t and position $\mathbf{r}$. D is an effective diffusion coefficient and k is an effective replication rate constant. The equation for the fractional seed concentration, $f(\mathbf{r},t)$, relative to the maximal seed concentration, eq. 1, is obtained by dividing the above by $P_{\max}$ and replacing $k = \kappa/P_{\max}$ and $P(\mathbf{r},t) = f(\mathbf{r},t)P_{\max}$. Fisher equation (i.e. the 1-dimensional version of eq. 1), for compact initial conditions such as a finite seed concentration at the origin, exhibits travelling wave solutions whose wave speed approaches $v = 2\sqrt{\kappa D}$.

We have not yet specified the dimensionality of the problem. Both a 3-dimensional model and a 1-dimensional model are reasonable: A 1-dimensional can be used as a simple representation of axonal spreading along one pathway, whereas a 3-dimensional one describes uniform spreading in space. More generally, in this problem the dimensionality simply encodes how much more space is available as seeds spread away from the origin.

Axonal spread on a branching network may best be described with a dimensionality between 1 and 3. Therefore, we here consider both extreme cases, 1-dimensional spreading and 3-dimensional spreading, and show that the qualitative results are the same. More importantly, in the replication-limited regime both models yield the same answer. As the accumulation of tau seeds in the data we analyse here occurs in the replication-limited regime, the rate constants we obtain from experimental measurements are thus independent of the dimensionality of the model chosen here.

In 3 dimensions we consider the spherically symmetric problem (so $\nabla^2 = \frac{1}{r^2}\frac{\partial^2}{\partial r^2}r^2$, where r is the distance from the origin) with an initial concentration of aggregates at the origin and the boundary condition of a solid wall at distance $r = r_{\max}$ (i.e. no flux through the surface at $r = r_{\max}$). The only difference to the 1 dimensional system is thus that $\nabla^2 = \frac{\partial^2}{\partial r^2}$.

$$\frac{\partial f(r,t)}{\partial t} = D\frac{1}{r^2}\frac{\partial}{\partial r^2}r^2 f(r,t) + \kappa f(r,t)\left[1 - f(r,t)\right] \tag{S2}$$

and in 1 dimension

$$\frac{\partial f(r,t)}{\partial t} = D\frac{\partial}{\partial r^2}r^2 f(r,t) + \kappa f(r,t)\left[1 - f(r,t)\right]. \tag{S3}$$

Limiting analytical solutions. In the replication-limit, the behaviour becomes independent of location, so an approximate solution to equation S2 can be obtained by setting the derivatives with respect to the spatial components to zero, yielding

$$f(\mathbf{r},t) = \frac{\bar{f}(0,t)e^{\kappa t}}{1 - \bar{f}(0,t) + \bar{f}(0,t)e^{\kappa t}}, \tag{S4}$$

where $\bar{f}(0,t)$ is the initial distribution, either averaged over all space if spreading is very fast, or simply the initial distribution if seeds are present everywhere and spreading is too slow to smooth out the initial distribution over the timescales of replication. This approximation is the same regardless of dimension.

By contrast, in the spreading limit, the initial profile of increase is propagated throughout the reaction volume with speed v. For the 1-dimensional Fisher equation, it has been shown that $v = 2\sqrt{D\kappa}$ for sufficiently sharp initial distributions. We will use this speed here to obtain an approximate solution in both the 1-dimensional and the 3-dimensional case. The approximate solution is thus

$$f(r,t) = \frac{f_0 e^{\kappa(t+r/v)}}{1 - f_0 + f_0 e^{\kappa(t+r/v)}}, \tag{S5}$$

where f_0 is the initial fraction of seeds at the origin. Note that this resembles equation (S4) with an additional time propagator term in the for of r/v. This approximation does not include the time delay due to diffusion of seeds from the initial distribution

to establish the shape of the propagating front, so it performs less well for sharp initial distributions. This solution does not feature in the data analysis of this work, we merely include it here for completion. More accurate solutions can be found in the extensive literature studying the Fisher equation. A comparison of these approximate solutions and the numerical integration of equation S2 is shown in Fig. S3.

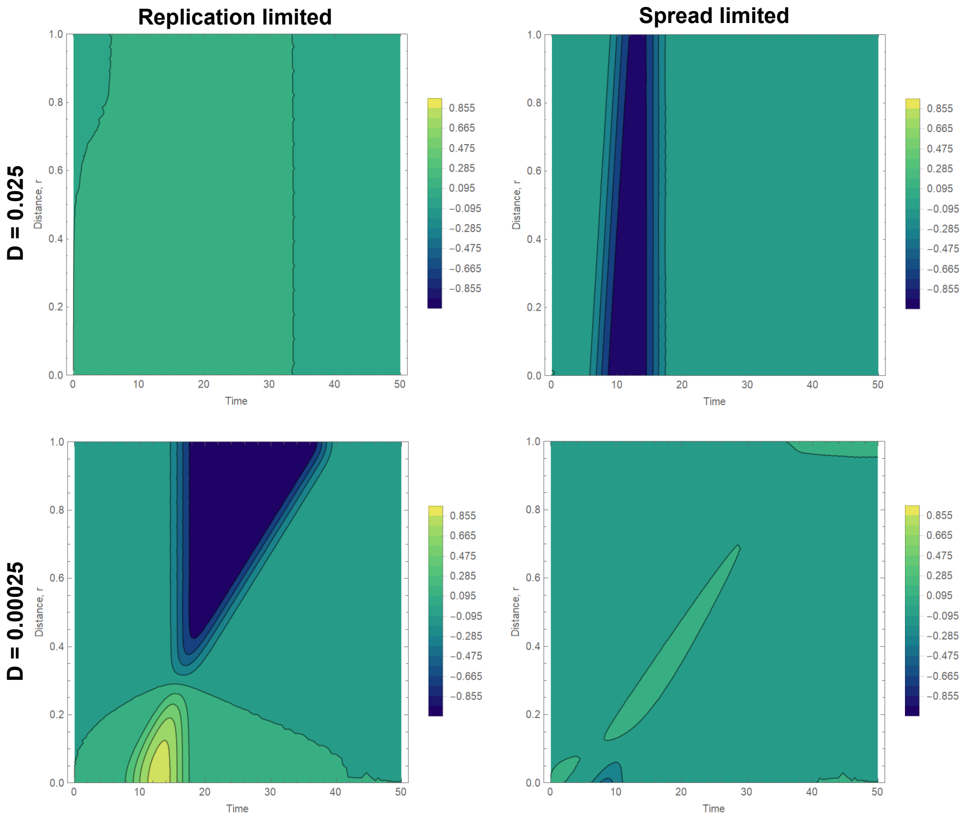

Figure S3: Difference between the numerical solution to equation S2 and the approximations (numerical - approximate), against distance and time. Green corresponds to good agreement, blue to an overestimation of the numerical solution and yellow to an underestimation of the numerical solution. The initial distribution was chosen to be $P(r, 0) = 0.1$ for $r < 0.01$ and 0 elsewhere. The approximation obtained in the replication limit, equation S4, performs well for fast diffusion $D/\kappa \gg 0.0025$, top row. The approximation obtained in the spreading limit, equation S5, performs better for slow diffusion $D/kappa \ll 0.0025$, reproducing the correct spreading velocity. However, the time taken to initially establish the travelling wave leads to a delay and slight offset of the numerical solution to our approximation.

Transition between regimes. A transition between the spreading-limited and replication-limited regimes can be said to occur when the time taken to spread throughout the entire

reaction volume is comparable to the time needed to reach the maximal aggregate concentration at any given point, i.e. when $r_{\max}/v \approx \tau_r$, where τ_r is the time-scale of the local reaction. We again approximate v by the speed of a travelling wave solution to the Fisher equation, $v \approx 2\sqrt{\kappa D}$, thus we obtain

$$\kappa D_{\text{switch}} \approx \frac{r_{\max}^2}{4\tau_r^2}. \tag{S6}$$

What remains is to estimate the time-scale of the local reaction τ_r. Using equation S4 to approximate the local behaviour, we obtain

$$\kappa\tau_r = \log\left(\frac{(1-f_{\text{init}})}{f_{\text{init}}}\right) + \log\left(\frac{f_{\text{end}}}{(1-f_{\text{end}})}\right), \tag{S7}$$

where f_{init} and f_{end} are the local concentrations at the beginning of the reaction and when it is considered completed, respectively. To estimate τ_r, these parameters need to be chosen. Their choice is determined by the specifics of the system, so we here choose it to resemble what we believe would be realistic scenarios. For reasonable values the exact choice has little effect on τ_r. We choose $P_{\text{end}} = 0.99$ and $P_{\text{init}} = 0.001$, giving a replication time-scale on the order of $\kappa\tau_r \approx 10$. Thus at the switch we have $\frac{D_{\text{switch}}}{\kappa} \approx 0.0025 r_{\max}^2$. Indeed, numerical integration of equation S2, does show the expected behaviour in the two limits and a switch at $D/\kappa \approx 0.0025 r_{\max}^2$, see main text Fig. 1.

2.2 Effect of initial distribution in each limit.

Here we illustrate the effect of lowering either the effective diffusion constant D or the replication rate κ by a factor of 3. While we label the limiting behaviours as spread-limited and replication-limited for simplicity, even in the spreading-limited regime, the velocity of the propagating front depends on the replication rate via $v \approx 2\sqrt{\kappa D}$, thus a decrease of κ always decreases the overall rate. We compare the case where the initial distribution is sharp and confined to a region close to the origin ($P(r,0) = 1$ for $r < 0.01$ and 0 otherwise) to the case where the initial distribution has most of its weight around the origin but has significant seed concentrations everywhere (modelling the distribution of seeds measured at Braak stage III).

For fast D, Figs. S4 and S5, reducing κ significantly reduces the rate at which new aggregates accumulate, regardless of initial distribution and dimensionality. Reducing D has little effect, except for a sharp initial distribution, where this decrease pushes the system more towards the transition to a spreading limited regime (if the simulations of the reference system were performed further away from this transition, this effect would disappear). The choice of dimensionality has a clear effect in this regime, mainly because the dimensionality determines what average concentration the initial distribution corresponds to. For example, the sharp initial distribution ($P(r,0) = 1$ for $r < 0.01$ and

0 otherwise) gives an average of $\langle P_0 \rangle = 1 * 0.1/1 = 0.1$ in 1 dimension but an average of $\langle P_0 \rangle = 1 * 0.1^3/1 = 0.001$ in 3 dimensions.

For slow D, Figs. S6 and S7, reducing κ decreases both the steepness and the speed of the propagating front and reducing D decreases the speed but increases the steepness for a sharp initial distribution. The 1-dimensional and the 3-dimensional systems behave very similarly. The effect of a decrease of either parameter is comparable, as expected. However, for the more spread out initial distribution, a decrease of κ significantly delays the reaction while a decrease of D has no apparent effect. This somewhat counter-intuitive observation originates in the fact that there are seeds present throughout the reaction volume already at early times: once the spreading rate decreases beyond a certain limit, the overall behaviour is again governed by the local replication of seeds.

In other words, at very high spreading rates the system becomes independent of the spreading because seeds are quickly distributed throughout the volume and the rate of this distribution does not limit the overall rate. However, if seeds are already present everywhere initially, the overall rate also becomes independent of the spreading rate at very low rates of spreading, because then the seeds already present locally replicate much quicker than new seeds can move into the region by spreading. Spreading is not required to allow this reaction to go to completion. The latter situation cannot arise for sharp distributions, where spreading is absolutely necessary because there are regions without any seeds, which can never reach completion without obtaining seeds by spread from other regions. In summary, while the overall rate in systems with a sharp initial distribution will depend on the spreading once the spreading rate is below a certain limit, the overall rate in systems with a more spread-out initial distribution is independent of the spreading both at low and high rates of spreading. The key conclusion are that

- In all cases, even in what we call a spreading-limited regime, reducing replication is the most effective strategy to prevent accumulation of aggregates.

- Spread-out distributions are never in a truly spreading limited regime.

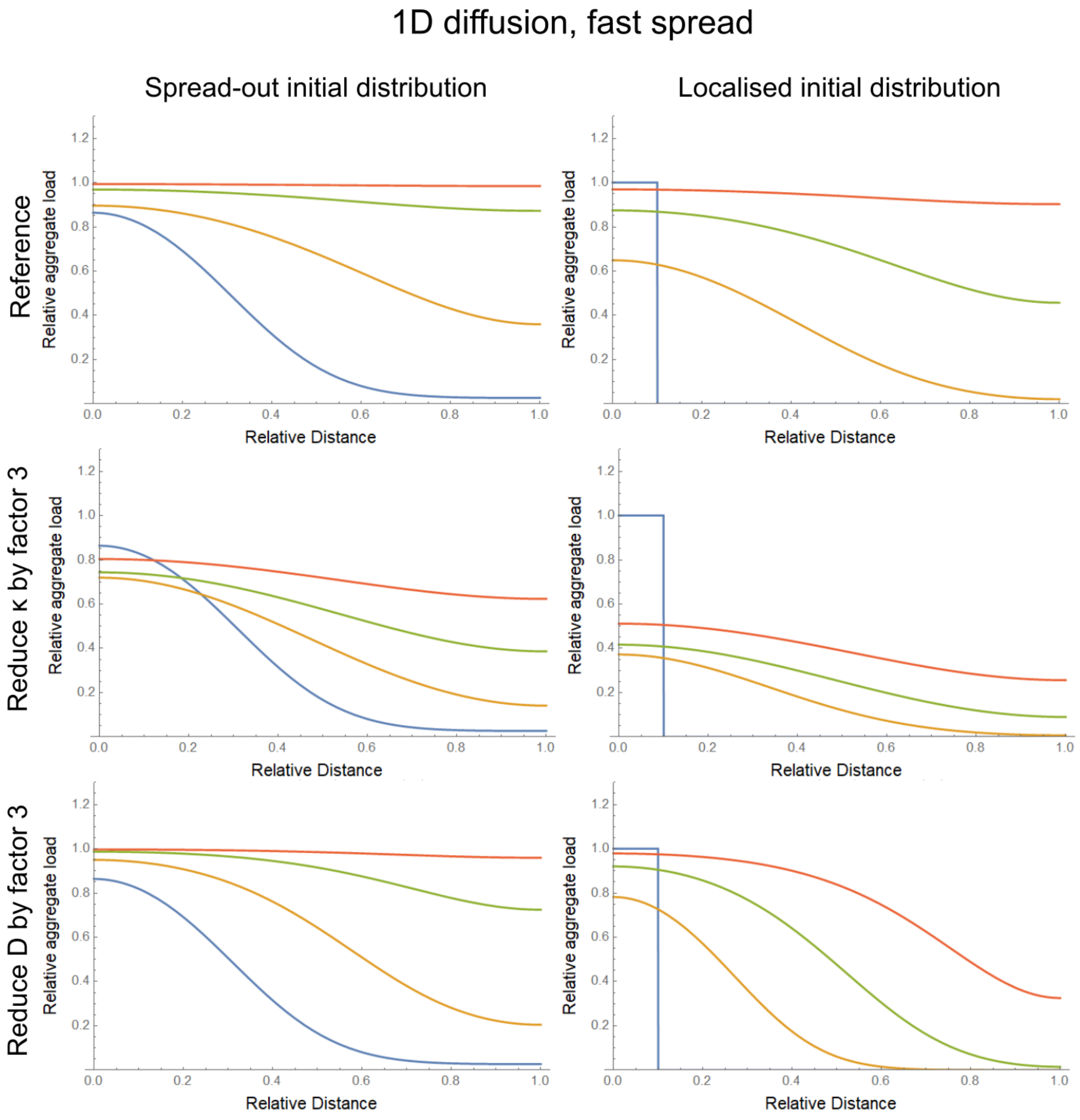

Figure S4: Effect of reduction in D or κ in the replication limited regime for 1-dimensional spreading, from numerical integration of equation S3. Reference values are $D = 0.025$, $\kappa = 1$ in reduced units. Times (0, 2, 4 and 6 in reduced units) are chosen to cover the range of behaviours.

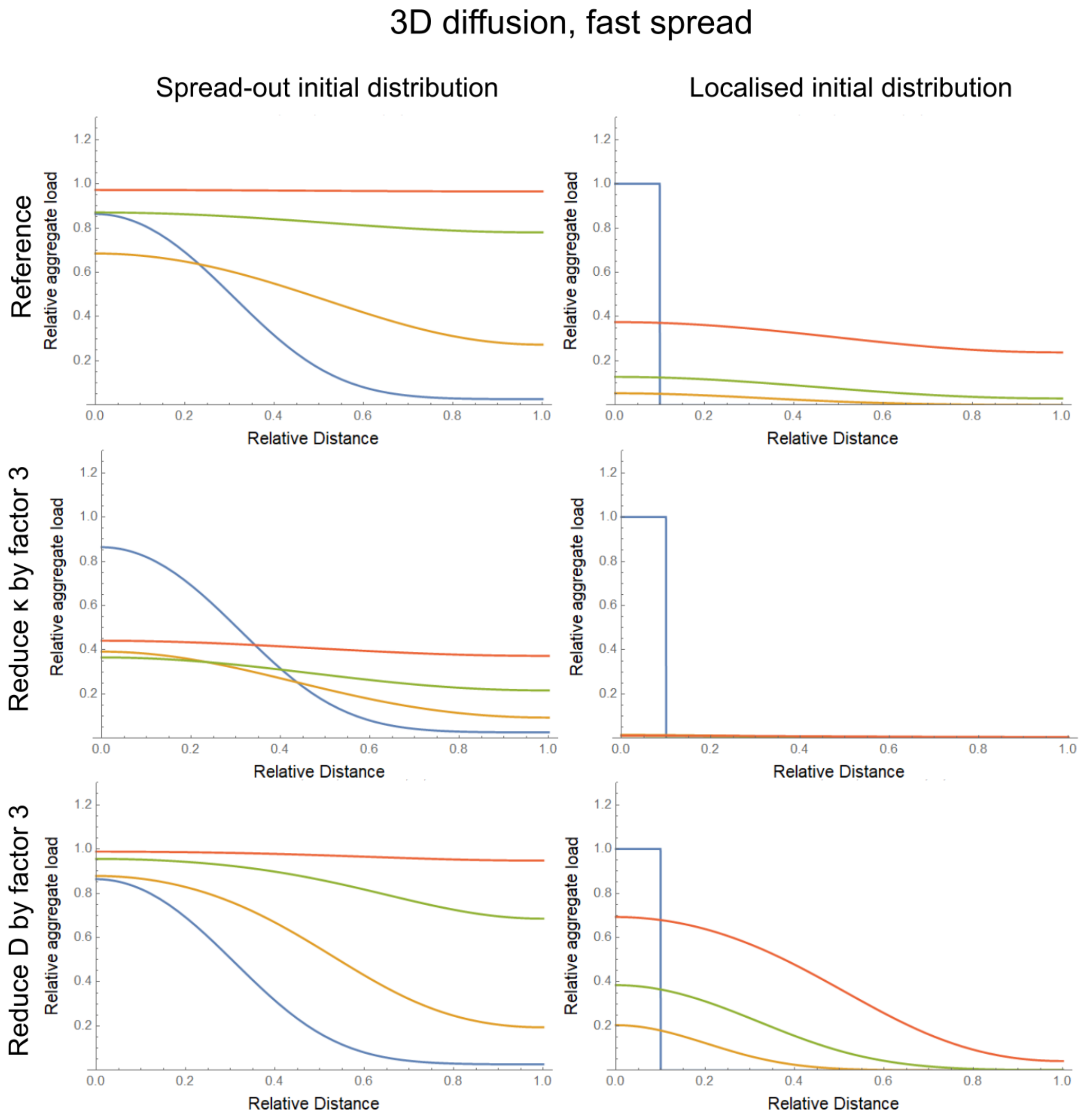

Figure S5: Effect of reduction in D or κ in the replication limited regime for 3-dimensional spreading, from numerical integration of equation S2. Reference values are $D = 0.025$, $\kappa = 1$ in reduced units. Times (0, 2, 4 and 6 in reduced units) are chosen to cover the range of behaviours.

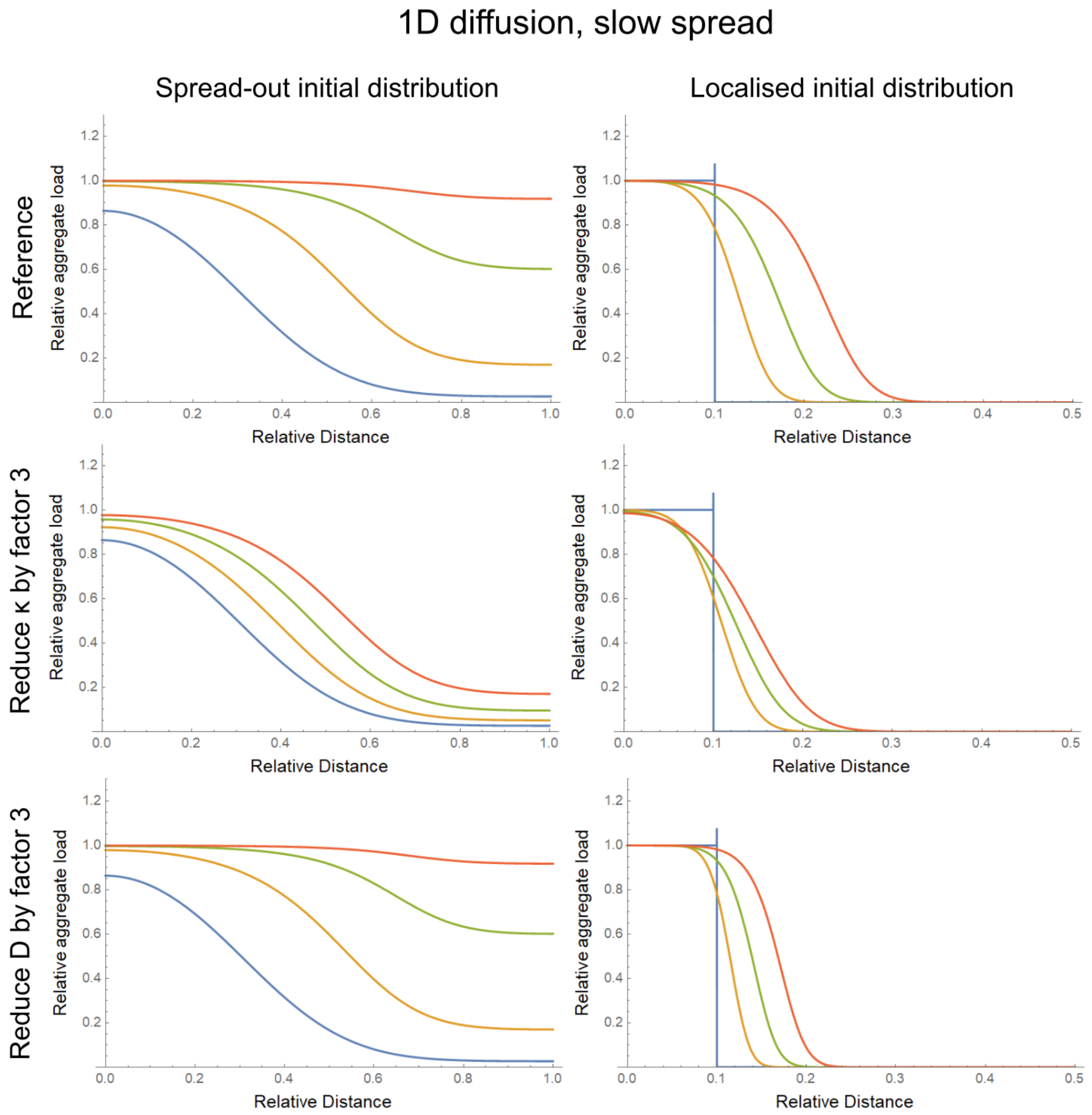

Figure S6: Effect of reduction in D or κ in the spreading limited regime for 1-dimensional spreading, from numerical integration of equation S3. Reference values are $D = 0.00025$, $\kappa = 1$ in reduced units. Times (0, 2, 4 and 6 in reduced units) are chosen to cover the range of behaviours.

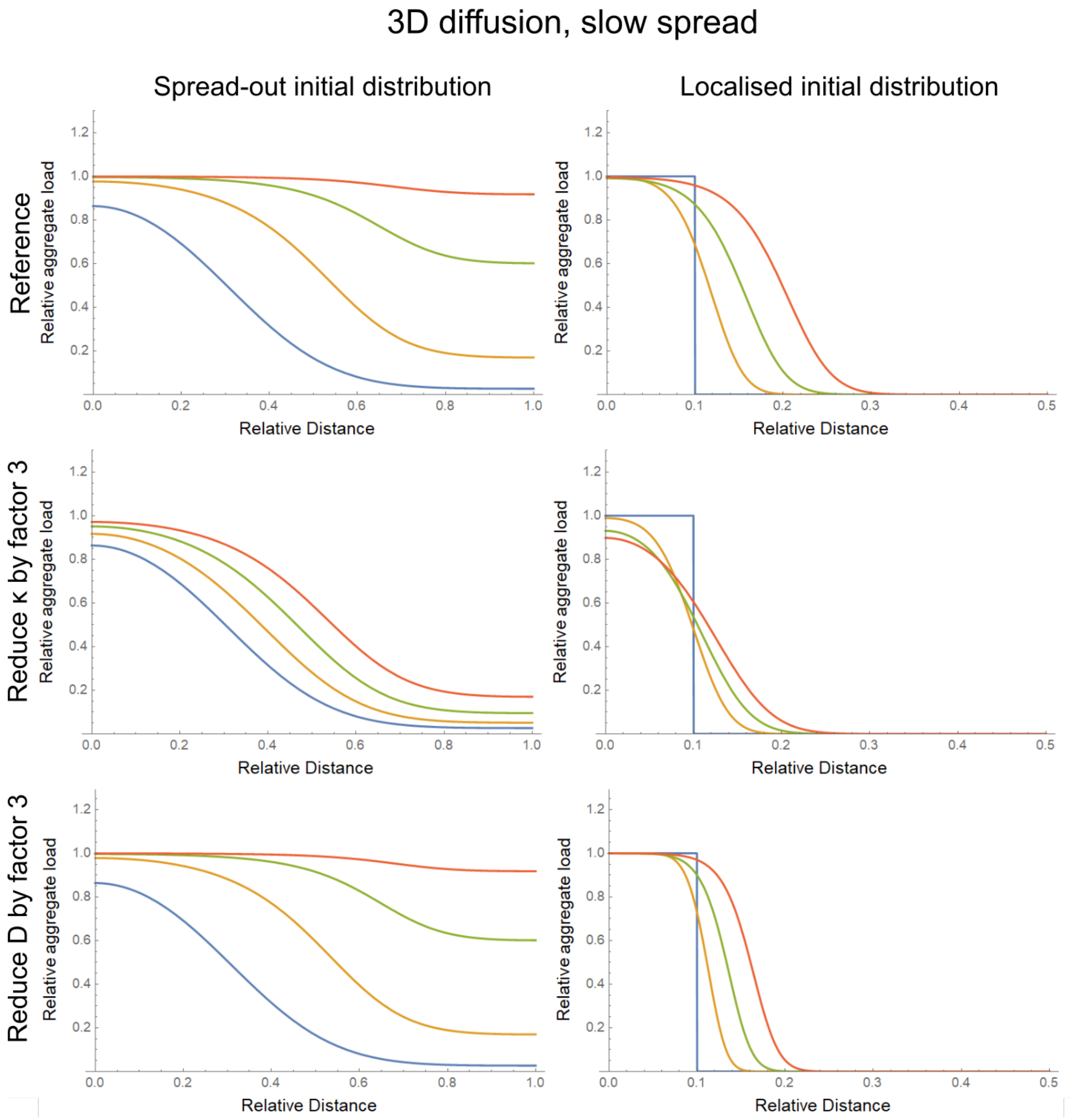

Figure S7: Effect of reduction in D or κ in the spreading limited regime for 3-dimensional spreading, from numerical integration of equation S2. Reference values are $D = 0.00025$, $\kappa = 1$ in reduced units. Times (0, 2, 4 and 6 in reduced units) are chosen to cover the range of behaviours.

2.3 Effect of dimensionality - Spread through space and along axons.

As shown in Figs. S4 and S5, starting from a Braak stage III distribution, both the 1D and the 3D models produce curves of a comparable shape. In both cases, we can conclude that the overall rate is limited by local replication, rather than spreading between brain regions. In reality, the brain is a much more connected network than our linear ordering of brain regions here would suggest. However, our simple model is sufficient to yield several basic principles, such as the emergence of different regimes, and the importance of replication in either regime, that will hold qualitatively even if the complexity of the underlying connectivity graph is increased significantly.

3 Relation to *in vitro* models.

Due to the wealth of data and high level of control over experimental conditions, such as the monomer concentrations, significantly more detailed mechanistic information can be extracted from measuring the aggregation of a purified protein *in vitro*. In particular, we are usually able to determine the detailed mechanism of aggregate multiplication. The in vivo data analysed here do not permit this level of detail to be extracted, however, the more detailed models we developed in the context of in vitro aggregation can be approximately mapped to the coarse-grained model used here. While the details of how all processes and their various extensions map onto the coarse-grained models used here is beyond the scope of this work, we give here some general guidelines on how the two can be related. A more detailed discussion can be found in Meisl *et al.* [37]. Spatial inhomogeneities are not generally considered in vitro, so we here simply compare the spatially independent version of equation S2 to the *in vitro* models. After some simplifications, the models of in vitro aggregation take the form of following non-linear coupled differential equations:

$$\frac{dP}{dt} = k_n m(t)^{n_c} + k_2 m(t)^{n_2} M(t) \tag{S8}$$

$$\frac{dM}{dt} = 2k_+ m(t) P(t), \tag{S9}$$

where $M(t)$ and $P(t)$ are the first and zeroeth moments of the fibril size distribution, i.e. the fibril mass and number concentrations, respectively and $m(t)$ is the free monomer concentration. The other parameters denote rate constants and reaction orders of the different processes on the pathway to aggregate formation. While the solutions of these equations generally take more complex forms than equation S4 derived here, they are generally similar in form, i.e. sigmoidal with an initially exponential increase, followed by a plateau region. *In vitro*, this plateau emerges from imposing conservation of mass via $M(t) + m(t) = m_{\text{tot}}$, *in vivo* it is an experimental observation and may arise due to a number of reasons, such as a decrease of protein synthesis in regions with high aggregate concentrations. For relating the models, this plateau region is of little importance and we instead focus on the early time exponential increase. A standard early time approximation *in vitro* is to set $m(t) = m_0$, as initially the depletion of monomer is negligible. This results in the following linear differential equations

$$\frac{dP}{dt} = k_n m_0^{n_c} + k_2 m_0^{n_2} M(t) \tag{S10}$$

$$\frac{dM}{dt} = 2k_+ m_0 P(t). \tag{S11}$$

Ignoring the contribution from the spontaneous formation of aggregates directly from monomer (the first term in the equation for dP/dt), the solutions are of the form

$$P(t) = P_0 \exp\left[\sqrt{2k_+ m_0 k_2 m_0^{n_2}} t\right]. \tag{S12}$$

Note that the exponential rate, $\sqrt{2k_+m_0k_2m_0^{n_2}}$ is the geometric mean of the rate of elongation, $2k_+m_0$, and the rate of multiplication, $k_2m_0^{n_2}$.

The early time solution for the equation we use here, i.e.

$$\frac{dP}{dt} = \kappa P(t)(1 - P(t)) \approx \kappa P(t), \tag{S13}$$

where κ is the replication rate, is given by

$$P(t) = P_0 \exp\left[\kappa t\right]. \tag{S14}$$

We therefore identify $\sqrt{2k_+m_0k_2m_0^{n_2}} \rightarrow \kappa$. In other words the replication rate here coarse-grained the processes of growth and multiplication into one rate and also subsumes their reaction orders and rate constants into one term. We can to some extent still dissect this replication rate by making use of the fact that growth and multiplication affect the average length differently, as we have done in the main part of this work, but the monomer dependence of processes is not yet accessible. Given the fact that many additional processes will be present *in vivo* that affect both multiplication and growth, the rates extracted from in vivo data represent effective rates of growth and multiplication rather than exact equivalents of their *in vitro* counterparts. However, to what extent growth or multiplication are affected points towards how these additional processes present *in vivo* affect aggregation.
